# A systematic review and meta-analysis of cognitive and behavioral tests in rodents treated with different doses of D-ribose

**DOI:** 10.3389/fnagi.2022.1036315

**Published:** 2022-11-09

**Authors:** Ying Song, Yage Du, Yu An, Jie Zheng, Yanhui Lu

**Affiliations:** ^1^School of Nursing, Peking University, Beijing, China; ^2^Department of Endocrinology, Beijing Chaoyang Hospital, Beijing, China

**Keywords:** D-ribose, rodent, cognition, behavioral test, meta-analysis

## Abstract

**Background:**

D-ribose is an aldehyde sugar and a necessary component of all living cells. Numerous reports have focused on D-ribose intervention in animal models to assess the negative effects of D-ribose on cognition. However, the results across these studies are inconsistent and the doses and actual effects of D-ribose on cognition remain unclear. This systematic review aimed to evaluate the effect of D-ribose on cognition in rodents.

**Methods:**

The articles from PubMed, Embase, Sciverse Scopus, Web of Science, the Chinese National Knowledge Infrastructure, SinoMed, Wanfang, and Cqvip databases were screened. The results from the abstract on cognitive-related behavioral tests and biochemical markers from the included articles were extracted and the reporting quality was assessed.

**Results:**

A total of eight trials involving 289 rodents met the eligibility criteria, and both low- and high-dose groups were included. Meta-analyses of these studies showed that D-ribose could cause a significant decrease in the number of platform crossings (standardized mean difference [SMD]: –0.80; 95% CI: –1.14, –0.46; *p* < 0.00001), percentage of distance traversed in the target quadrant (SMD: –1.20; 95% CI: –1.47, –0.92; *p* < 0.00001), percentage of time spent in the target quadrant (SMD: –0.93; 95% CI: –1.18, –0.68; *p* < 0.00001), and prolonged escape latency (SMD: 0.41; 95% CI: 0.16, 0.65; *p* = 0.001) in the Morris water maze test. Moreover, D-ribose intervention increased the levels of advanced glycation end products (AGEs) in the brain (SMD: 0.49; 95% CI: 0.34, 0.63; *p* < 0.00001) and blood (SMD: 0.50; 95% CI: 0.08, 0.92; *p* = 0.02). Subsequently, subgroup analysis for the dose of D-ribose intervention revealed that high doses injured cognitive function more significantly than low D-ribose doses.

**Conclusion:**

D-ribose treatment caused cognitive impairment, and cognition deteriorated with increasing dose. Furthermore, the increase in AGEs in the blood and brain confirmed that D-ribose may be involved in cognitive impairment through non-enzymatic glycosylation resulting in the generation of AGEs. These findings provide a new research idea for unveiling basic mechanisms and prospective therapeutic targets for the prevention and treatment of patients with cognitive impairment.

## Introduction

Alzheimer’s disease (AD) is the most common type of dementia, accounting for 60–80% of all reported cases (Yankner, 1996; Abramov and Duchen, 2005). AD is a progressive neurodegenerative disease characterized by cognitive deficits, irreversibly destroying memory, language, thinking, and other important mental skills (Devadhasan et al., 2011; Ashraf et al., 2015, 2018; Lee et al., 2017). The estimates from the World Health Organization suggest that approximately 50 million individuals suffered from AD, with nearly 10 million new cases being added annually. Globally, the number of elderly is increasing rapidly, making AD a critical public health concern in the 21st century (Dartigues, 2009; Scheltens et al., 2021). According to the Alzheimer Report 2022, the overall global cost of dementia treatment is more than US$800 billion. Jia et al. (2018) estimated that the cost was US$957.56 billion in 2015 and is expected to rise to US$2.54 trillion in 2030, and US$9.12 trillion in 2050. The public health system is also heavily burdened by AD (Alzheimers Dementia, 2021). However, specific risk factors and mechanisms underlying AD remain unclear.

D-ribose is an aldehyde sugar present in all living cells and plays significant biological roles (Akhter et al., 2016; Li et al., 2021). It is a component of RNA and adenosine triphosphate (ATP) and a synthetic material for nucleotide coenzymes and vitamin B2 (Broom et al., 1964; Keller et al., 1988; Wei et al., 2012). It can be synthesized endogenously from glucose through the pentose phosphate pathway and exogenously from riboflavin-rich foods like fruits and vegetables (Mauser et al., 1985; Dhanoa and Housner, 2007). Serum D-ribose level in humans is 0.02 mM in healthy people, and 0.01–0.1 mM in the cerebrospinal fluid (Seuffer, 1977; Cai et al., 2005). In the case of metabolic diseases, including diabetes mellitus, the serum and urinary D-ribose levels are elevated, resulting in the dysregulation of D-ribose metabolism. Evidence shows that dysregulated D-ribose metabolism causes several neurodegenerative diseases such as AD.

As a reactive sugar, D-ribose can bind to protein or lipid molecules for non-enzymatic glycation, resulting in the production of advanced glycation end products (AGEs) (Wei et al., 2012; Akhter et al., 2014; Siddiqui et al., 2018). Reports show that AGEs are involved in the pathogenesis of aging, diabetes, and neurodegenerative diseases (Akhter et al., 2013, 2015). Moreover, AGEs are neurotoxic in cultured neurons, and their precursors, including methylglyoxal and glyoxal, also promote intracellular aggregation of amyloid-beta carboxy-terminal fragments and cytotoxicity (Takeuchi et al., 2000; Woltjer et al., 2003). An animal study also reported that D-ribose accelerated the formation of AGEs in astrocytes, ultimately activating the NF-κB pathway in the brain and causing cognitive impairment (Han et al., 2014). Chen et al. (2019b) showed that the synthesis of hepatic triglycerides is significantly influenced by D-ribose, which in turn affects hepatocellular steatosis and cognitive functions.

Animal experiments show that D-ribose impairs the spatial learning and memory of mice. For example, Han et al. (2014) demonstrated that high concentrations of D-ribose led to cognitive losses in mice. Notably, not all studies suggest that D-ribose significantly affects spatial learning and memory, especially those using low-dose D-ribose intervention. For example, Han et al. (2011) found that low concentrations of D-ribose showed no effects on spatial cognition. The inconsistent results might be due to the differences in the intervention doses of D-ribose. To date, D-ribose and its role in cognition remain unclear. Therefore, we systematically reviewed and analyzed the available evidence to clarify the actual effects of D-ribose and its differential doses on cognition.

## Methods

### Literature search and screen

A literature search on the effects of D-ribose on cognitive dysfunction was performed using the search term, “ribose,” in combination with “Cognitive Dysfunction, Cognition Disorders, Dementia, AD, Cognitive Impairment, Mild Cognitive Impairment, Mild Neurocognitive Disorder, Cognitive Decline, and Memory deficits,” in PubMed, Embase, Sciverse Scopus, Web of Science, the Chinese National Knowledge Infrastructure (CNKI), SinoMed, Wanfang, and Cqvip databases for studies published from database creation up to January 2022. In the beginning, no filters were applied for the search, including language, publication date, dose of administration, route of administration, and duration of administration. Two reviewers (SY and YD) examined the papers to ensure that they met the inclusion criteria for the meta-analysis. A third member (YL) was consulted in case of a disagreement.

### Eligibility criteria

The systematic review was limited to published studies, i.e., random assignment of rodents to treatment groups. An intact design with at least one healthy control group of rodents treated with vehicle (saline, phosphate-buffered saline, or a similar solution) and at least an experimental group of rodents treated with D-ribose was an inclusion criterion. All the included studies reported at least one measure of learning and memory after the intervention. Studies with incomplete data in the published text or Supplementary material, or in cases of D-ribose administered with other components, were excluded.

### Quality assessment and data extraction

The Animal Research: Reporting *in vivo* Experiments (ARRIVE) guidelines checklist 2.0 including 21 entries was used to assess the quality of each study (Percie et al., 2020). Two reviewers (SY and YD) independently extracted the data in a standardized format suited for animal study design from the reports that met the inclusion criteria. The items recorded were as follows: fundamental information (name of the author, year of publication, and nation); animals (animal species, age, weight, gender, and sample size); study design (dose, route, and duration of D-ribose manipulation); and measurement outcomes (behavioral test results such as those of the Morris water maze test and biochemistry such as AGEs). If data were not obtained in a table or in text, the Web Plot Digitizer software to extract data from images was used to obtain these from the published figures (Drevon et al., 2017). For studies that compared the results of groups treated with different D-ribose doses with those of a single control group, the data from the latter were utilized in each of the dose intervention meta-analyses. The mean score and standard deviation [SD] were extracted for the meta-analysis. If an error was presented as a standard error [SE] or a 95% CI, the formula provided in the Cochrane Handbook was used to convert it into SD values.

### Statistical analysis

Data were analyzed using the Review Manager (version 5.4, The Nordic Cochrane Centre, The Cochrane Collaboration, Copenhagen, Denmark). According to the Cochrane Handbook for Systematic Reviews of Interventions, the standardized mean difference (SMD) corresponding to the difference of the means of the two groups and 95% CIs as our effect size of interest was used to estimate the effect of D-ribose treatment on cognitive outcomes (García-Bonilla et al., 2012). The *I*^2^ statistic was used to assess heterogeneity, reflecting the percentage variance between trials for low (<50%) and high (≥50%) heterogeneity values. A random effect model was used when heterogeneity was more than 50%, and a fixed effect model was employed when heterogeneity was less than 50% (Borenstein et al., 2010). A value of α = 0.05 was considered statistically significant. Subgroup analysis was conducted for the dose of D-ribose treatment (low dose: <1 g/kg/day vs. high dose: ≥2 g/kg/day). To assess the possible causes for heterogeneity in case of a significant difference (*I*^2^≥ 50%), sensitivity analysis was performed by removing the included data points one by one to see whether the changes had any impact on the combined result estimate.

## Results

### Study selection and inclusion

**FIGURE 1:**
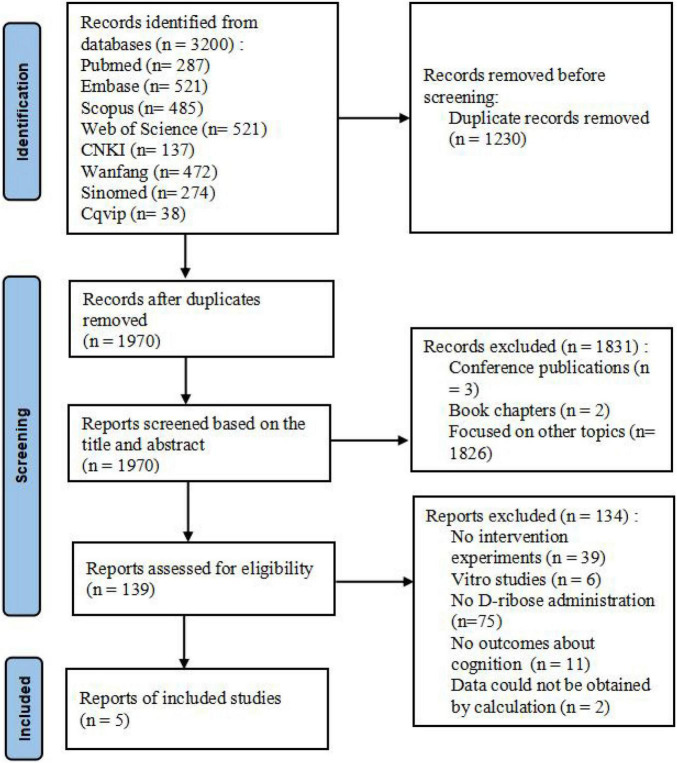
Prisma flowchart depicting the article selection process.

A total of 3,200 reports were identified including 287 from PubMed, 521 from Embase, 485 from Scopus, 986 from Web of Science, 137 from CNKI, 472 from Wanfang, 274 from SinoMed, and 38 from Cqvip. Subsequently, 1,970 records were retained after all the searches were pooled and duplicates were deleted. After construing all the titles and abstracts, 1,831 records were found to be irrelevant (the majority of these were excluded as they were conference publications or studies focused on other topics). After reading the full text of the remaining 139 records, 5 studies met the predefined eligibility criteria. Non-consensus on the inclusion of these articles was not applicable. The representation of the article selection process is shown in Figure 1.

### Study characteristics

Most of the included studies were published in the last decade, and 2 were published in the past 3 years. Among the 5 studies included, C57BL/6J mice were used in 4 reports, while Sprague Dawley rats were used in the remaining one. All the male rodent models were 8–10 weeks old and no study reported the animal weights.

For the D-ribose treatment, the most frequently selected duration was 30 days (2 studies), followed by 10 days (1 study), 28 days (1 study), and 180 days (1 study). Intraperitoneal injection was the most commonly used route for D-ribose administration, except for two studies, which employed intravenous tail injection and gavage administration, respectively. Among the total of 11 experimental groups in the 5 studies, 6 used a dose of less than 1 g/kg/day; 4 employed a dose of more than 2 g/kg/day, and 1 used a dose of 1.6 g/kg/day according to administration protocol. The specific characteristics of the included studies are shown in Table 1.

**TABLE 1:** Characteristics of the included studies.

References	Country/ Region	Animals						Intervention		
			
		Species	Age	Weight	Gender	Sample size		Way	Dose	Duration (day)
						Intervention group	Control group			
Xu et al., 2021	China	C57BL/6J mice	8–10 weeks	\	Male	Low-dose *n* = 15 high-dose *n* = 22	*n* = 22	Intraperitoneal injection	Low-dose 0.4 g/kg/day	28
									High-dose 4 g/kg/day	
Wu et al., 2019	China	Sprague Dawley rats	8 weeks	\	Male	*n* = 10	*n* = 10	Tail intravenous injection	0.025 g/kg/day	30
Han et al., 2011	China	C57BL/6J mice	8–10 weeks	\	Male	Low-dose *n* = 12 high-dose *n* = 12	*n* = 12	Intraperitoneal injection	Low-dose 0.2 g/kg/day	30
									High-dose 2 g/kg/day	
Wu et al., 2015	China	C57BL/6J mice	8 weeks	\	Male	Low-dose *n* = 12 high-dose *n* = 12	*n* = 12	Gavage administration	Low-dose 0.375 g/kg/day	180
									High-dose 3.75 g/kg/day	
Han et al., 2014	China	C57BL/6J mice	8–10 weeks	\	Male	*n* = 12	*n* = 12	Intraperitoneal injection	First dose 0.4 g/kg/day	10
									Second dose 0.8 g/kg/day	
									Third dose 1.6 g/kg/day	
									Forth dose 3.2 g/kg/day	

### Study quality

In the ARRIVE guidelines for assessing the quality of studies, all showed the four items, comprising the title, objectives, experimental outcomes, and estimated outcomes. For the experimental procedure, although most studies mentioned, “What was done, how it was done, and what was used” and “When and how often,” only two studies detailed “Why (provided the rationale for procedures).” All studies presented the sample size but they did not explain how the sample size was estimated. The specific results of the quality assessment were shown in Table 2.

**TABLE 2:** Reporting the quality of the included studies.

Study	ARRIVE guideline																			
	
	Introduction						Methods							Results				Discussion		
				
	Title	Ab-stract	Back-ground	Objec-tives	Ethical state-ment	Study design	Experi-mental proce-dure	Experi-mental animals	Hous-ing/Hus-bandry	Sample size	Allo-cating animals to experi-mental groups	Experi-mental out-comes	Statis-tical methods	Base-line data	Num-bers ana-lyzed	Out-comes and esti-mation	Adverse events	Interpre-tation/Scientific impli-cations	Generaliz-ability/Transla-tion	Fund-ing
Ke Xu et al.	F	P	F F	F	F	F F F F	P P F P	P N	F F N	F N NA	P N F	F	F F N	F	F NA	F	N N	F F N	F	F
Beibei Wu et al.	F	P	F P	F	F	F P F N	P P F N	P F	F P N	F N NA	P N N	F	P F N	F	F N	F	N N	F F N	P	F
Chanshuai Han et al.	F	F	F P	F	F	F P F N	P P F N	P F	F N N	F N N	P N N	F	P F N	F	F N	F	N N	F P N	F	F
Beibei Wu et al.	F	F	F F	F	F	F P F N	F P F F	P F	F N N	F N N	P N N	F	P F N	F	F N	F	N N	F P N	N	F
C Han et al.	F	P	F N	F	F	F P F N	P P F N	P F	F N N	F N N	P N N	F	P F N	F	F N	F	N N	F N N	P	F

ARRIVE, animal research: Reporting in vivo experiments; F, fully reported; P, partially reported; N, not reported; NA, not applicable.

### Meta-analysis of study outcomes

#### Morris water maze test

All studies described the effects of D-ribose on cognition in the Morris water maze test, and these studies were included in the meta-analysis. Three studies reported the number of platform crossings; four studies reported the percentage of the distance traversed in the target quadrant; five studies reported the percentage of time spent in the target quadrant, and all studies reported the escape latency.

**FIGURE 2:**
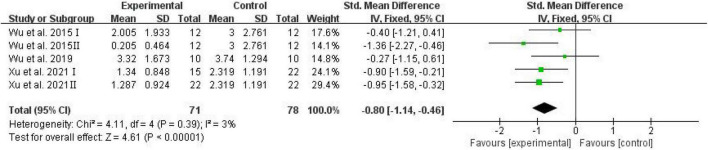
Forest plot of the number of platforms crossing in the Morris water maze test.

**FIGURE 3:**
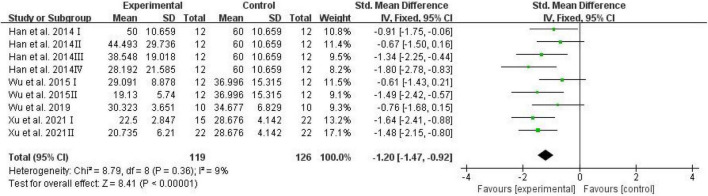
Forest plot of the percentage of distance in target quadrant in the Morris water maze test.

**FIGURE 4:**
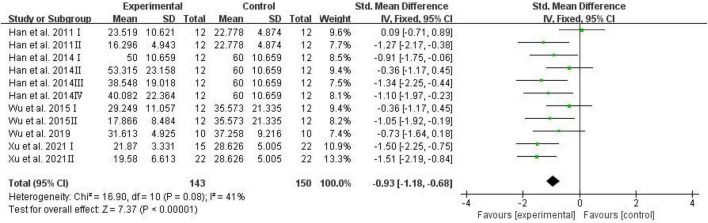
Forest plot of the percentage of time in target quadrant in the Morris water maze test.

The number of platform crossings, the percentage of the distance traversed in the target quadrant, and the percentage of time spent in the target quadrant were counted and analyzed by RevMan in trials to test memory functions (Vorhees and Williams, 2006). Meta-analysis indicated that D-ribose reduced the number of platform crossings (Figure 2; SMD: –0.80; 95% CI: –1.14, –0.46; *p* < 0.00001) with less heterogeneity among studies (*I*^2^ = 3%). Moreover, in a total of 245 animals, the meta-analysis showed less percentage of the distance traversed in the target quadrant in the D-ribose-treated group as compared to the control group, with an SMD of –1.20, suggesting less heterogeneity (Figure 3; 95% CI: –1.47, –0.92; *p* < 0.00001; *I*^2^ = 9%). The D-ribose group showed a substantially decreased percentage of time spent in the target quadrant relative to the control group (Figure 4; SMD: –0.93; 95% CI: –1.18, –0.68; *p* < 0.00001). Evidence of low heterogeneity among studies was found (*I*^2^ = 41%). The heterogeneity in the sensitivity analysis obviously declined after excluding the data in the low-dose group in the study reported by Han et al. (2011) (*I*^2^ = 9%).

**FIGURE 5:**
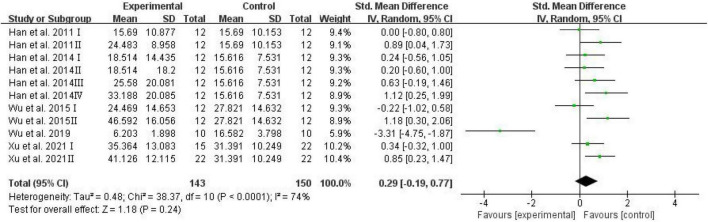
Forest plot of the escape latency in the Morris water maze test.

The escape latency was assessed and analyzed by RevMan in trials to test spatial learning ability (Vorhees and Williams, 2006). Meta-analysis revealed that D-ribose intervention had no significant effects on escape latency (Figure 5; SMD: 0.29; 95% CI: –0.19, 0.77; *p* = 0.24).

#### Levels of advanced glycation end products

**FIGURE 6:**
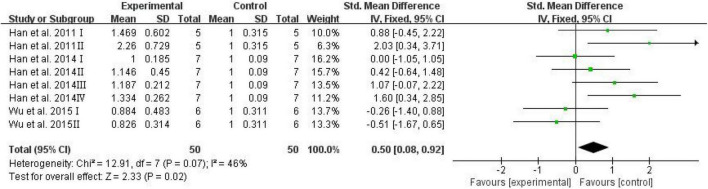
Forest plot of the serum AGEs.

Advanced glycation end products have been investigated extensively owing to their involvement in cognitive diseases such as AD (Vlassara et al., 1994; Dukic-Stefanovic et al., 2001). Serum AGEs were adopted as an outcome in three studies, and the analysis showed the effects of D-ribose in significantly elevating AGEs in mouse blood (Figure 6; SMD: 0.50; 95% CI: 0.08, 0.92; *p* = 0.02); the heterogeneity in the studies was acceptable (*I*^2^ = 46%), and the results remained unaffected after sensitivity analysis.

**FIGURE 7:**
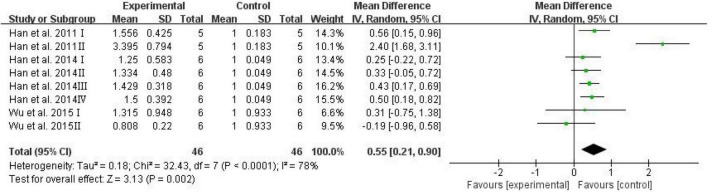
Forest plot of the brain AGEs.

The brain AGEs were adopted as an outcome in three studies, and D-ribose intervention significantly increased the levels of AGEs in the mouse brain (Figure 7; SMD: 0.55; 95% CI: 0.21, 0.90; *p* = 0.002). However, there was significant heterogeneity among the studies (*I*^2^ = 78%). The heterogeneity in the sensitivity analysis completely disappeared after excluding the data of the high-dose group in the study reported by Han et al. (2011) (*I*^2^ = 0%).

### Subgroup analysis

To further investigate the potential influence of dose on the effects of D-ribose on cognition and assess the source of heterogeneity, a subgroup analysis was conducted based on the dose of D-ribose intervention (low dose: <1 g/kg/day vs. high dose: ≥2 g/kg/day).

After the D-ribose intervention, the number of platform crossings reduced remarkably in both groups of D-ribose intervention, and the group with a high intervention dose had a larger effect size (SMD = –1.08; 95% CI: –1.60, –0.57; *p* < 0.0001) as compared to the group with a low administration of D-ribose dose (SMD = –0.58; 95% CI: –1.03, –0.13; *p* = 0.01). Similarly, regarding the percentage of the distance traversed and time spent in the target quadrant, the effect was more pronounced in the group with a high D-ribose dose intervention (distance: SMD = –1.56; 95% CI: –2.03, –1.08; *p* < 0.00001; time: SMD = –1.27; 95% CI: –1.68, –0.87; *p* < 0.00001). In addition, D-ribose intervention showed no significant influence on escape latency. However, the subgroup analysis revealed that the escape latency increased significantly in the group with a high D-ribose dose intervention (SMD = 0.98; 95% CI: 0.59, 1.37; *p* < 0.00001), while the group with a low D-ribose dose intervention did not affect the escape latency in rodents (SMD = –0.29; 95% CI: –1.01, 0.43; *p* = 0.44).

The specific results of the subgroup analysis are shown in Table 3. Subgroup analysis showed that the levels of serum AGEs increased markedly in the group with a high D-ribose dose intervention (SMD = 0.27; 95% CI: 0.10, 0.44; *p* = 0.002), while there was no significant difference in the group with a low D-ribose dose intervention as compared to the controls (SMD = 0.03; 95% CI: –0.10, 0.16; *p* = 0.60). Moreover, the brain AGEs reduced significantly in both groups; however, a larger effect size was found in the group with a high D-ribose dose (SMD = 0.69; 95% CI: 0.42, 0.96; *p* < 0.00001) as compared to the group with a low D-ribose dose intervention (SMD = 0.39; 95% CI: 0.15, 0.62; *p* = 0.001).

**TABLE 3:** Results of subgroup analysis.

Results	Subgroups	Estimate mean (95% CI)	Heterogeneity	Studies (n)	Comparisons (n)
The number of platform crossing	High dose	SMD = –1.08; 95% CI: –1.60, –0.57	*I*^2^ = 0%; *p* = 0.46	34	34
	Low dose	SMD = –0.58; 95% CI: –1.03,-0.13	*I*^2^ = 0%; *p* = 0.48	34	44
The percentage of distance in target quadrant	High dose	SMD = –1.56; 95% CI:-2.03,-1.08	*I*^2^ = 0%; *p* = 0.85	46	46
	Low dose	SMD = –0.95; 95% CI: –1.32, –0.58	I^2^ = 9%;p = 0.35	61	68
The percentage of time in target quadrant	High dose	SMD = –1.27; 95% CI:-1.68,-0.87	*I*^2^ = 0%; *p* = 0.83	58	58
	Low dose	SMD = –0.64; 95% CI: –0.97, –0.31	I^2^ = 48%;p = 0.09	73	80
The escape latency	High dose	SMD = 0.98; 95% CI: 0.59,1.37	*I*^2^ = 0%; *p* = 0.92	58	58
	Low dose	SMD = –0.29; 95% CI: –1.01, 0.43	I^2^ = 77%;p = 0.0005	73	80
Serum AGEs	High dose	SMD = 0.27; 95% CI:0.10,0.44	*I*^2^ = 86%; *p* = 0.0008	18	18
	Low dose	SMD = 0.03; 95% CI: –0.10, 0.16	I^2^ = 2%;p = 0.38	25	25
Brain AGEs	High dose	SMD = 0.69; 95% CI:0.42,0.96	*I*^2^ = 93%; *p* < 0.00001	17	17
	Low dose	SMD = 0.39; 95% CI: 0.15, 0.62	I^2^ = 0%;p = 0.78	23	23

## Discussion

Metabolic disorder of ribose is associated with adverse effects on cognition (Siddiqui et al., 2018). Although studies focused on D-ribose intervention show inconsistent findings, no systematic review of D-ribose on cognitive alterations has been published. We examined animal studies to assess the effects of D-ribose on cognition through a systematic review and meta-analysis of the impact of differential D-ribose doses on cognition.

D-ribose treatment caused cognitive impairment, and the cognition deteriorated with increasing dose. We assessed the effects of different doses of D-ribose on cognitive changes in mice and rats according to the results of the Morris water maze, the number of platform crossings, the percentage of the distance traversed in the target quadrant, and the percentage of time spent in the target quadrant to test memory function and escape latency to assess spatial learning abilities. The meta-analysis revealed that D-ribose intervention produced a significant impairment in the spatial learning task but not in the spatial memory task. We verified that D-ribose caused cognitive impairment, consistent with previous studies, which suggest that metabolic disorders due to D-ribose are a possible risk factor for age-related neurodegenerative disorders such as AD (Zhu et al., 2022). Lyu et al. (2019) found that D-ribose levels in patients with AD were considerably higher than those in age-matched controls with normal cognition. A cross-sectional study reported that T2DM-MCI patients had higher serum concentrations of D-ribose and were correlated negatively with the MoCA score (Lu et al., 2021).

In addition, our subgroup analysis revealed that the group with a high D-ribose dose intervention injured cognitive function more significantly than the group with a low D-ribose dose intervention. It was clear that the impaired spatial memory ability was more prominent in the high-dose group than the low-dose D-ribose-treated group. D-ribose at a low dose did not cause a decline in the spatial learning ability in rodents, and only D-ribose at a high dose led to a significant decrease in the spatial learning ability. For the increase in brain AGEs, D-ribose at a high dose had a stronger impact relative to the low-dose D-ribose group. Moreover, only a high dose of D-ribose but not a low dose led to the rise in AGEs in serum. These outcomes clearly suggested cognitive detriment due to D-ribose at a high dose.

Sensitivity analysis explained most of the heterogeneity. In terms of the significant reduction of heterogeneity in the percentage of time spent in the target quadrant, contrary results were found in the study of Han et al. (2011), whereby a low dose of D-ribose was used. Similarly, the obvious reduction of heterogeneity in the latency was attributed to the results reported by Wu et al. (2019) which were contrary to other studies. Regarding the brain AGEs, sensitivity analysis revealed the source of heterogeneity which may be attributed to differences in dose, sample size, and eligibility criteria (Garcia-Alamino et al., 2017).

In the included studies, the rodents used were all male. Population-based studies suggest that gender may affect cognitive impairment (Au et al., 2017; Tang et al., 2019; C Silva et al., 2022). Nonetheless, owing to hormonal secretion, physical fitness, and other factors, males are usually selected as experimental subjects in animal studies (Schaeffer, 2018). Hence, future research is needed to further address these gender-based differences. As for sample size, due to ethical and economic reasons, a sample size calculation is not necessary for each experiment, and at least 7–10 animals are utilized per group in most animal studies (Festing, 2018; Ricci et al., 2020). The sample size can be further calculated based on power analysis, precision analysis, and other methods in future studies.

Evidence suggests that D-ribose is involved in the generation of free oxygen radicals, glycation, protein aggregation, AGEs, and age-related neurodegenerative illnesses (Chen et al., 2009, 2017, 2019a; Batkulwar et al., 2018). According to cell-based experiments, D-ribose interacts with proteins and produces AGEs. Furthermore, the expression of the receptor of advanced glycation end products (RAGE) is linked to AGE elevation caused by ribosylation in both astrocytoma cells and astrocytes, resulting in RAGE-dependent NF-κB activation and astrocyte stimulation, further impairing the spatial learning and memory (Han et al., 2014). Our analysis of AGEs is consistent with the conclusions reported previously, i.e., ribose-induced cognitive impairment may be related to AGEs produced by non-enzymatic glycosylation of D-ribose (Chen et al., 2010; Wei et al., 2012). These findings suggested that D-ribose-induced non-enzymatic glycosylation may play a role in the pathogenesis of cognitive impairment. However, the mechanisms underlying D-ribose-mediated cognitive impairment remain unclear, and these should be further investigated in the future.

## Limitations

This review, however, has some limitations, including a relatively small total sample size and the number of included studies. Furthermore, poor reporting quality increased the likelihood of bias and reduced the validity of the findings. To validate the harm caused by D-ribose in cognitive impairment and comprehensively study the underlying mechanisms, more precise and rigorous experiments with high sample sizes are warranted.

## Conclusion

We summarized the effects of D-ribose intervention with different doses based on cognitive and behavioral tests and found that D-ribose was related to learning and memory functions. Our findings indicate that D-ribose intervention causes cognitive impairment, and cognition deteriorated with increasing dose. Furthermore, the increase in AGEs in the blood and brain confirmed that D-ribose may be involved in cognitive impairment through glycosylation, resulting in the generation of AGEs. These provide a new research direction for unveiling basic mechanisms and prospective therapeutic targets for the prevention and treatment of cognitive impairment in these patients.

## Data availability statement

The raw data supporting the conclusions of this article will be made available by the authors, without undue reservation.

## Author contributions

YS and YD completed the data analysis and wrote the manuscript. JZ contributed to the data analysis. YL and YA supervised the project. All authors reviewed and approved the submitted version.

## References

[B1] Abramov A. Y. Duchen M. R. (2005). The role of an astrocytic NADPH oxidas e in the neurotoxicity of amyloid beta peptides. *Philos. Trans. R. Soc. Lond. B Biol. Sci.* 360 2309–2314. 10.1098/rstb.2005.1766 16321801 PMC1569597

[B2] Akhter F. Khan M. S. Alatar A. A. Faisal M. Ahmad S. (2016). Antigenic role of the adaptive immune response to d-ribose glycated LDL in diabetes, atherosclerosis and diabetes atherosclerotic patients. *Life Sci.* 151 139–146. 10.1016/j.lfs.2016.02.013 26874030

[B3] Akhter F. Khan M. S. Ahmad S. (2015). Acquired immunogenicity of calf thymus DNA and LDL modified by D-ribose: a comparative study. *Int. J. Biol. Macromol.* 72 1222–1227. 10.1016/j.ijbiomac.2014.10.034 25450543

[B4] Akhter F. Khan M. S. Singh S. Ahmad S. (2014). An immunohistochemical analysis to validate the rationale behind the enhanced immunogenicity of D-ribosylated low density lipo-protein. *PLoS One* 9:e113144. 10.1371/journal.pone.0113144 25393017 PMC4231124

[B5] Akhter F. Salman Khan M. Shahab U. Moinuddin Ahmad S. (2013). Bio-physical characterization of ribose induced glycation: a mechanistic study on DNA perturbations. *Int. J. Biol. Macromol.* 58 206–210. 10.1016/j.ijbiomac.2013.03.036 23524157

[B6] Alzheimers Dementia (2021). 2021 Alzheimer’s disease facts and figures. *Alzheimers Dement.* 17 327–406. 10.1002/alz.12328 33756057

[B7] Ashraf G. M. Tabrez S. Jabir N. R. Firoz C. K. Ahmad S. Hassan I. (2015). An overview on global trends in nanotechnological approaches for Alzheimer therapy. *Curr. Drug Metab.* 16 719–727. 10.2174/138920021608151107125757 26560324

[B8] Ashraf J. M. Ansari M. A. Fatma S. Abdullah S. M. S. Iqbal J. Madkhali A. (2018). Inhibiting effect of zinc oxide nanoparticles on advanced glycation products and oxidative modifications: A potential tool to counteract oxidative stress in neurodegenerative diseases. *Mol. Neurobiol.* 55 7438–7452. 10.1007/s12035-018-0935-x 29423819

[B9] Au B. Dale-McGrath S. Tierney M. C. (2017). Sex differences in the prevalence and incidence of mild cognitive impairment: A meta-analysis. *Ageing Res. Rev.* 35 176–199. 27771474 10.1016/j.arr.2016.09.005

[B10] Batkulwar K. Godbole R. Banarjee R. Kassaar O. Williams R. J. Kulkarni M. J. (2018). Advanced glycation end products modulate amyloidogenic APP processing and tau phosphorylation: A mechanistic link between glycation and the development of Alzheimer’s disease. *ACS Chem. Neurosci.* 9 988–1000. 10.1021/acschemneuro.7b00410 29384651

[B11] Borenstein M. Hedges L. V. Higgins J. P. Rothstein H. R. (2010). A basic introduction to fixed-effect and random-effects models for meta-analysis. *Res. Synth. Methods* 1 97–111. 10.1002/jrsm.12 26061376

[B12] Broom A. D. Townsend L. B. Jones J. W. Robins R. K. (1964). Purine nucleosides.6. Further methylation studies of naturally occurring purine nucleosides. *Biochemistry* 3 494–500. 14188163 10.1021/bi00892a005

[B13] C Silva T. Zhang W. Young J. I. Gomez L. Schmidt M. A. Varma A. (2022). Distinct sex-specific DNA methylation differences in Alzheimer’s disease. *Alzheimers Res. Ther.* 14:133. 10.1186/s13195-022-01070-z 36109771 PMC9479371

[B14] Cai Y. Liu J. Shi Y. Liang L. Mou S. (2005). Determination of several sugars in serum by high-performance anion-exchange chromatography with pulsed amperometric detection. *J. Chromatogr. A* 1085 98–103. 10.1016/j.chroma.2004.11.100 16106854

[B15] Chen L. Wei Y. Wang X. He R. (2009). D-Ribosylated Tau forms globular aggregates with high cytotoxicity. *Cell. Mol. Life Sci.* 66 2559–2571. 10.1007/s00018-009-0058-7 19517062 PMC11115892

[B16] Chen L. Wei Y. Wang X. He R. (2010). Ribosylation rapidly induces alpha-synuclein to form highly cytotoxic molten globules of advanced glycation end products. *PLoS One* 5:e9052. 10.1371/journal.pone.0009052 20140223 PMC2816216

[B17] Chen X. Su T. Chen Y. He Y. Liu Y. Xu Y. (2017). d-Ribose as a Contributor to Glycated Haemoglobin. *EBioMedicine* 25 143–153. 10.1016/j.ebiom.2017.10.001 29033370 PMC5704047

[B18] Chen Y. Yu L. Wei Y. Long Y. Xu Y. He T. (2019b). D-ribose increases triglyceride via upregulation of DGAT in the liver. *Sci. China Life Sci.* 62 858–861. 10.1007/s11427-019-9542-2 31049804

[B19] Chen Y. Yu L. Wang Y. Wei Y. Xu Y. He T. (2019a). d-Ribose contributes to the glycation of serum protein. *Biochim. Biophys. Acta Mol. Basis Dis.* 1865 2285–2292. 10.1016/j.bbadis.2019.05.005 31085227

[B20] Dartigues J. F. (2009). Alzheimer’s disease: a global challenge for the 21st century. *Lancet Neurol.* 8 1082–1083. 19909903 10.1016/S1474-4422(09)70298-4

[B21] Devadhasan J. P. Kim S. An J. (2011). Fish-on-a-chip: a sensitive detection microfluidic system for Alzheimer’s disease. *J. Biomed. Sci.* 18:33. 10.1186/1423-0127-18-33 21619660 PMC3125339

[B22] Dhanoa T. S. Housner J. A. (2007). Ribose: more than a simple sugar? *Curr. Sports Med. Rep.* 6 254–257. 17618002 10.1007/s11932-007-0041-8

[B23] Drevon D. Fursa S. R. Malcolm A. L. (2017). Intercoder reliability and validity of webplotdigitizer in extracting graphed data. *Behav. Modif.* 41 323–339. 10.1177/0145445516673998 27760807

[B24] Dukic-Stefanovic S. Schinzel R. Riederer P. Münch G. (2001). AGES in brain ageing: AGE-inhibitors as neuroprotective and anti-dementia drugs? *Biogerontology* 2 19–34. 10.1023/a:1010052800347 11708614

[B25] Festing M. F. (2018). On determining sample size in experiments involving laboratory animals. *Lab. Anim.* 52 341–350. 29310487 10.1177/0023677217738268

[B26] Garcia-Alamino J. M. Bankhead C. Heneghan C. Pidduck N. Perera R. (2017). Impact of heterogeneity and effect size on the estimation of the optimal information size: analysis of recently published meta-analyses. *BMJ Open* 7:e015888. 10.1136/bmjopen-2017-015888 29122784 PMC5695413

[B27] García-Bonilla L. Campos M. Giralt D. Salat D. Chacón P. Hernández-Guillamon M. (2012). Evidence for the efficacy of statins in animal stroke models: A meta-analysis. *J. Neurochem.* 122 233–243. 22548274 10.1111/j.1471-4159.2012.07773.x

[B28] Han C. Lu Y. Wei Y. Liu Y. He R. (2011). D-ribose induces cellular protein glycation and impairs mouse spatial cognition. *PLoS One* 6:e24623. 10.1371/journal.pone.0024623 21966363 PMC3169629

[B29] Han C. Lu Y. Wei Y. Wu B. Liu Y. He R. (2014). D-ribosylation induces cognitive impairment through RAGE-dependent astrocytic inflammation. *Cell Death Dis.* 5:e1117. 10.1038/cddis.2014.89 24625976 PMC3973213

[B30] Jia J. Wei C. Chen S. Li F. Tang Y. Qin W. (2018). The cost of Alzheimer’s disease in China and re-estimation of costs worldwide. *Alzheimers Dement.* 14 483–491. 10.1016/j.jalz.2017.12.006 29433981

[B31] Keller P. J. Le Van Q. Kim S. U. Bown D. H. Chen H. C. Kohnle A. (1988). Biosynthesis of riboflavin: mechanism of formation of the ribitylamino linkage. *Biochemistry* 27 1117–1120. 10.1021/bi00404a006 3130093

[B32] Lee D. Lee W.-S. Lim S. Kim Y. K. Jung H.-Y. Das S. (2017). A guanidine-appended scyllo-inositol derivative AAD-66 enhances brain delivery and ameliorates Alzheimer’s phenotypes. *Sci. Rep.* 7:14125. 10.1038/s41598-017-14559-7 29074878 PMC5658413

[B33] Li S. Wang J. Xiao Y. Zhang L. Fang J. Yang N. (2021). D-ribose: Potential clinical applications in congestive heart failure and diabetes, and its complications (Review). *Exp. Ther. Med.* 21:496. 10.3892/etm.2021.9927 33791005 PMC8005739

[B34] Lu Y. Jiang H. Zhang H. Li R. Zhang Q. Luo D. (2021). Serum oxidized low density lipoprotein serves as a mediator for the inverse relationship between serum D-ribose and cognitive performance in type 2 diabetic patients. *Free Radic. Biol. Med.* 171 91–98. 10.1016/j.freeradbiomed.2021.05.015 33989757

[B35] Lyu J. Yu L. X. He Y. G. Wei Y. Rong-Qiao H. (2019). A Brief Study of the Correlation of Urine D-ribose with MMSE Scores of Patients with Alzheimer’s Disease and Cognitively Normal Participants. *Am. J. Urol. Res.* 4 18–23.

[B36] Mauser M. Hoffmeister H. M. Nienaber C. Schaper W. (1985). Influence of ribose, adenosine, and “AICAR” on the rate of myocardial adenosine triphosphate synthesis during reperfusion after coronary artery occlusion in the dog. *Circ. Res.* 56 220–230. 10.1161/01.res.56.2.220 3918804

[B37] Percie D. S. N. Hurst V. Ahluwalia A. Alam S. Avey M. T. Baker M. (2020). The ARRIVE guidelines 2.0: Updated guidelines for reporting animal research. *PLoS Biol.* 18:e3000410. 10.1371/journal.pbio.3000410 32663219 PMC7360023

[B38] Ricci C. Baumgartner J. Malan L. Smuts C. M. (2020). Determining sample size adequacy for animal model studies in nutrition research: limits and ethical challenges of ordinary power calculation procedures. *Int. J. Food Sci. Nutr.* 71 256–264. 10.1080/09637486.2019.1646714 31379222

[B39] Schaeffer L. R. (2018). Necessary changes to improve animal models. *J. Anim. Breed Genet.* 135 124–131. 29575102 10.1111/jbg.12321

[B40] Scheltens P. Blennow K. Breteler M. M. de Strooper B. Frisoni G. B. Salloway S. (2021). Alzheimer’s disease. *Lancet* 397 1577–1590. 26921134 10.1016/S0140-6736(15)01124-1

[B41] Seuffer R. (1977). [A new method for the determination of sugars in cerebrospinal fluid (author’s transl)]. *J. Clin. Chem. Clin. Biochem.* 15 663–668. 604418

[B42] Siddiqui Z. Ishtikhar M. Moinuddin Ahmad S. (2018). d-Ribose induced glycoxidative insult to hemoglobin protein: An approach to spot its structural perturbations. *Int. J. Biol. Macromol.* 112 134–147. 29378270 10.1016/j.ijbiomac.2018.01.161

[B43] Takeuchi M. Bucala R. Suzuki T. Ohkubo T. Yamazaki M. Koike T. (2000). Neurotoxicity of advanced glycation end-products for cultured cortical neurons. *J. Neuropathol. Exp. Neurol.* 59 1094–1105. 11138929 10.1093/jnen/59.12.1094

[B44] Tang F. Chi I. Dong X. (2019). Sex differences in the prevalence and incidence of cognitive impairment: Does immigration matter? *J. Am. Geriatr. Soc.* 67 S513–S518. 31403204 10.1111/jgs.15728

[B45] Vlassara H. Bucala R. Striker L. (1994). Pathogenic effects of advanced glycosylation: Biochemical, biologic, and clinical implications for diabetes and aging. *Lab. Invest.* 70 138–151. 8139257

[B46] Vorhees C. V. Williams M. T. (2006). Morris water maze: Procedures for assessing spatial and related forms of learning and memory. *Nat. Protoc.* 1 848–858. 17406317 10.1038/nprot.2006.116 PMC2895266

[B47] Wei Y. Han C. S. Zhou J. Liu Y. Chen L. He R. Q. (2012). D-ribose in glycation and protein aggregation. *Biochim. Biophys. Acta* 1820 488–494. 22274132 10.1016/j.bbagen.2012.01.005

[B48] Woltjer R. L. Maezawa I. Ou J. J. Montine K. S. Montine T. J. (2003). Advanced glycation endproduct precursor alters intracellular amyloid-beta/A beta PP carboxy-terminal fragment aggregation and cytotoxicity. *J. Alzheimers Dis.* 5 467–476. 14757937 10.3233/jad-2003-5607

[B49] Wu B. Wei Y. Wang Y. Su T. Zhou L. Liu Y. (2015). Gavage of D-Ribose induces A -like deposits, Tau hyperphosphorylation as well as memory loss and anxiety-like behavior in mice. *Oncotarget* 6, 34128–34142. 26452037 10.18632/oncotarget.6021 PMC4741441

[B50] Wu B. Wang Y. Shi C. Chen Y. Yu L. Li J. (2019). Ribosylation-derived advanced glycation end products induce tau hyperphosphorylation through brain-derived neurotrophic factor reduction. *J. Alzheimers Dis.* 71 291–305. 31381511 10.3233/JAD-190158

[B51] Xu K. Wang M. Zhou W. Pu J. Wang H. Xie P. (2021). Chronic D-ribose and D-mannose overload induce depressive/anxiety-like behavior and spatial memory impairment in mice. *Transl. Psychiatry* 11:90. 10.1038/s41398-020-01126-4 33531473 PMC7854712

[B52] Yankner B. A. (1996). Mechanisms of neuronal degeneration in Alzheimer’s disease. *Neuron* 16 921–932. 8630250 10.1016/s0896-6273(00)80115-4

[B53] Zhu X. Zhao C. Liu J. Qin F. Xiong Z. Zhao L. (2022). Urine D-ribose levels correlate with cognitive function in community-dwelling older adults. *BMC Geriatr.* 22:693. 10.1186/s12877-022-03288-w 35996093 PMC9396817

